# Inhibitory effect of anti-Scg3 on corneal neovascularization: a preliminary study

**DOI:** 10.1186/s12886-022-02690-7

**Published:** 2022-11-28

**Authors:** He Jin, Binbin Yang, Dongdong Jiang, Zhixiang Ding, Yu Xiong, Xinsheng Zeng

**Affiliations:** 1 Affiliated Hospital of Guilin Medical University, Guilin Medical University, 541001 Guilin, China; 2 Nanxishan Hospital of Guangxi Zhuang Autonomous Region, Guilin Medical University, 541001 Guilin, China

**Keywords:** Corneal neovascularization, Secretogranin III, Vascular growth factor, Corneal alkali burn

## Abstract

**Background:**

Corneal neovascularization (CNV) is an important disease that causes blindness. Secretogranin III (Scg3) has emerged as a new influencing factor of neovascularization. This study analyzed the Scg3 antibody’s inhibitory effect on CNV and and explored its preliminary mechanism.

**Methods:**

Human umbilical vein endothelial cells (HUVECs) were treated with Scg3 and anti-Scg3. Cell proliferation, wound healing migration and tube formation assays were performed. Healthy adult New Zealand rabbits were randomly selected to be alkali burned and establish the corneal neovascularization (CNV) model. The rabbits were randomly divided into 3 groups (the high concentration group, low concentration group and control group). Different doses of anti-Scg3 and PBS were administered to the rabbits. Clinical examinations, immunostaining, quantitative real-time polymerase chain reaction (qPCR) and western blotting analyses were performed postoperatively.

**Results:**

In the in vitro study, the Scg3 antibody mixture inhibited Scg3-induced endothelial cell proliferation and angiogenesis. In the in vivo study, significant CNV was observed in the control group. Confocal microscopy also revealed considerable active neovascularization in the control group. There was no obvious CNV growth in the high concentration group. Additionally, CD31, LYVE1 and CD45 expression was significantly inhibited after treatment with a high concentration of Scg3 antibody. The qPCR and western blotting analyses revealed that the levels of ERK in the low concentration group and high concentration group were higher than those in the control group at 7 days and 14 days. The levels of VEGF in the control group were significantly increased compared with those in the high concentration group. In all three groups, the levels of Akt were not significantly different at any time point.

**Conclusion:**

The expression of Scg3 could affect the growth of HUVECs in vitro. Treatment with a high concentration (0.5 µg/mL) of Scg3 antibody reduced the inflammatory response and inhibited the growth of corneal neovascularization after corneal alkali burn injury in rabbits. The MEK/ERK pathway might play an important role in the inhibitory effect of anti-Scg3.

**Supplementary Information:**

The online version contains supplementary material available at 10.1186/s12886-022-02690-7.

## Introduction

Corneal disease ranks third in the world’s major causes of blindness [1]. Corneal neovascularization (CNV) is an important disease that causes blindness [2]. Corneal neovascularization can be caused by ocular chemical damage, trauma, keratitis, or long-term incorrect use of contact lenses. In our previous study, we also found that CNV was a major factor that caused corneal graft rejection and transplantation failure [3, 4]. These different kinds of stimulation lead to the growth of limbal neovascularization into the transparent corneal area, resulting in corneal oedema and corneal opacity [2]. The obvious corneal opacity and corresponding irregular astigmatism caused by refractive changes are the main causes of vision loss [5, 6].

There are a variety of treatments for corneal neovascularization, such as laser photocoagulation, photodynamic therapy, anti-VEGF medication and surgery [7].these treatments can inhibit the growth of corneal neovascularization to a certain extent [8–10], but they also have some therapeutic side effects. laser photocoagulation and photodynamic therapy only could cause temporary occlusion and withdrawal to relieve symptoms [11]. Notara et al. found that the high concentration of bevacizumab would downregulate the integrin α and β, and which interferes with the adhesion mechanism of cell migration and affects corneal epithelial healing [12], resulting in thinning of the corneal stroma [13]. Yin et al. found the CNV progressed in the discontinuation of bevacizumab sometimes [14]. And we also evaluated the effect of ranibizumab (10 mg/ml) and conbercept (10 mg/ml) in the rabbit alkali burn CNV model in our initial study. We found the CNV was stimulated caused by aggravation of the inflammatory response [15, 16]. We still need the evaluation of degree of burn and drug concentration to verify the validity of these results. The surgery always used the amniotic membrane, and amniotic membranes’ instability would affect the effectiveness of amniotic membrane. Therefore, no clear consensus has been reached on the most efficacious treatment option for CNV, which indicates that novel treatments for CNV diseases are in urgent need [7].

Many studies [17] have shown that many vascular growth factors are involved in CNV formation. Li’s study [18] found that Secretogranin III (Scg3) and VEGF exerted similar effects, and both could affect the growth of neovascularization in diabetic retinopathy. Therefore, Scg3 has emerged as a new influencing factor of neovascularization [18]. Scg3 is a granin family member that contains chromaffin A, pheochromogranin B, and secretory proteins II-VII [19]. Granin family could form regulatory hormones and growth factors by stimulating the large dense core vesicle, and these hormones and factors take participate in the regulation of various biological activities [20–22]. Additionally, employing the revolutionary technology of comparative ligandomics, Scg3 was recently demonstrated to be a novel angiogenesis growth factor in a mouse model of diabetic retinopathy [18]. However, its effect on the CNV model and the underlying molecular mechanism remain poorly understood.

In our previous study, we established a rabbit model of alkali burns to study corneal neovascularization. We have found the Scg3 expression in this rabbit CNV model [23]. And with Scg3 antibodies interference, the levels of the animal tears’ inflammatory cytokines(ICAM-1, IL-10 and TGF-β1) were decreased [23]. In this study, we administered Scg3 antibodies to the rabbit CNV model to evaluate whether the Scg3 antibody could effectively inhibit the formation of corneal neovascularization and to provide new signaling pathways corneal neovascularization.

## Methods

### Study design

In the in vitro study, human umbilical vein endothelial cells (HUVECs) were cultured with Scg3 and anti-Scg3. Cell proliferation, wound healing migration and tube formation assays were performed. For the in vivo study, we established a rabbit model of alkali burns. Different doses (0.1 µg/mL and 0.5 µg/mL) of anti-Scg3 were administered to the rabbits. We observed the ocular appearance of the rabbits. Immunofluorescence staining of neovascularization, leukocyte’s and lymphatic markers was performed. Additionally, the expression levels of neovascularization markers and signaling pathway markers were measured at the gene and protein levels.

### Materials and animals

Human umbilical vein endothelial cells (HUVECs) were purchased from Procell (Wuhan, CN). Human Scg3 was purchased from Sino Biological. Anti-Scg3 antibodies were purchased from LifeSpan (USA). The Ethics Committee at Guilin Medical University authorized all experimental procedures (Acceptance number: 2019-0005). This study utilized eighty mature New Zealand white rabbits. All animals were housed in a clean room with a controlled atmosphere in a facility approved by the Accreditation of Laboratory Animal Care (Animal Experiment Center of Guilin Medical University). Additionally, during the study, the animals were housed individually and given free access to food and water.

### In vitro study

In the cell proliferation assay, HUVECs were seeded at 5000 cells/well in 96-well plates precoated with Attachment Factor. The cells were treated with Scg3 (1 µg/ml) alone, Scg3 (1 µg/ml) and anti-Scg3 (2 µg/ml) or PBS control alone at 37 °C in 5% CO_2_. The experiment was conducted in at least five replicate wells. A total of 10 µL of Cell Counting Kit (CCK-8) reagent (CK04, Dojindo Laboratories) was added to the cells at the 12, 24 and 48 h time points and incubated for another 2 h at 37 °C in 5% CO_2_. The optical density (OD) value of each well was measured at a wavelength of 450 nm on a microplate reader.

HUVEC were seeded in pre-coated 6-well plates, cultivated to 90–100% confluence, and starved for 3 h in DMEM supplemented with 0.2% serum for the wound healing migration assay. Scratch lines were created using 200-µl pipette tips. The floating cells were removed with PBS. The cells were further incubated with Scg3 (1 µg/ml) alone, Scg3 (1 µg/ml) and anti-Scg3 (2 µg/ml) or PBS control alone in DMEM supplemented with 2% FBS for an additional 20 h. At 0 and 20 h, images were obtained using a light microscope (before and after growth factor treatment). The closure of the wound was determined by the number of cells that moved to the ablated area.

In the tube formation assay, 96-well plates were precoated with Matrigel (50 µl/well) (356,234, Corning), which was allowed to solidify at 37 °C for 30 min. HUVECs were starved in DMEM supplemented with 0.2% FBS overnight. Cells were harvested, resuspended, and seeded on Matrigel (15,000 cells/well), further incubated with Scg3 (1 µg/ml) alone, Scg3 (1 µg/ml) and anti-Scg3 (2 µg/ml) or PBS control alone in DMEM supplemented with 2% FBS for 7 h at 37 °C in 5% CO_2_. Phase-contrast microscopy was used to measure the length of the manifold, the number of tubes, and the branch points in the cells.

### Corneal angiogenesis assay

The rabbits were anaesthetized using an intramuscular injection of serazine hydrochloride (0.2 mg/kg). The right eye was chosen to be subjected to alkali burns. Circular filter paper with a diameter of 8 mm was soaked in 1 mol/L sodium hydroxide solution for 10 s. This filter paper was placed in the middle of the rabbit’s cornea for 40 s. Then, the cornea subjected to alkali burn was fully washed with 0.9% sodium chloride solution for 3 min. Levofloxacin eye drops were administered once after alkali burn to prevent infection.

The rabbits were randomly separated into 3 groups (the low concentration group, the high concentration group and the control group) following alkali burn. The low concentration group received 0.1 mL Scg3 antibody (0.1 µg/ml) subconjunctivally, while the high concentration group received 0.1 mL Scg3 antibody (0.5 µg/ml). The rabbits in the control group received 0.1 mL of 1× PBS subconjunctivally. Subconjunctival injection was given in the right eye of all the rabbits. And subconjunctival injection was given once every four days from the alkali burn day, and the rabbits were observed for 14 days. Corneal angiogenesis was evaluated using a slit-lamp microscope and confocal microscopy.

### Immunofluorescence assay

The corneas were fixed in optimal cutting temperature compound (OCT Compound, SAKURA, Japan) and then frozen overnight at -20 °C. The sections were prepared at a thickness of 6 μm. Then, 3% H_2_O_2_ was used to block the corneas for 15 min, and 10% sera (matched to the secondary antibodies species) was used to block the corneas for 20 min. The sections were incubated with the primary antibodies (CD31, 1:500, NOVUS, NB100-64796SS; CD45, 1:200, Gene Tex, GTX116018; and LYVE-1, 1:100, Abcam, ab14917) overnight at 4 °C and then incubated with FITC-conjugated secondary antibodies for 30 min at room temperature. After counterstaining with 4,6-diamidino-2-phenylin-dole (DAPI) for 5 min, these cells were subsequently observed under a fluorescence microscope (AxioCam MRc, ZEISS) at 40× magnification.

### Quantitative real-time polymerase chain reaction (qPCR)

A grinding rod was used to crush the cornea in liquid nitrogen, and then, total RNA was extracted using TRLZOL reagent (Ambion, Invitrogen). Two micrograms of total RNA was reverse transcribed to cDNA using MonScript RTIII All-in-One Mix (Monad, CN) according to the manufacturer’s instructions. Quantitative real-time PCR (qPCR) was performed using MonAmp™ Green qPCR Mix (Monad, CN). The mRNA expression levels of vascular endothelial growth factor (VEGF), extracellular signal-regulated kinase (ERK1/2) and Akt were measured with the ACI 7500HT sequence detection system (Thermo, USA). The qPCR program was as follows: 30-second initial denaturation at 95°C, 40 cycles of 10-second denaturation at 95°C and 30-second annealing, and a final extension at 60°C. Complementary forward and reverse primers were designed as follows: VEGF (forward: 5’- GATGAAAGCCTGGAGTGCGT − 3’; reverse: 5’- TCATCTCCCCTATGTGCTGG − 3’), Akt (forward: 5’- GAAAACCAGTCCTCCACGAAC − 3’; reverse: 5’- TTCCAGGTAGAAGGGTCCGT − 3’), ERK1/2 (forward: 5’- GTCCAGGCAACAAAAGAGGTG − 3’; reverse: 5’- GCAGGTGATCGTAGGTTCTAGC − 3’), glyceraldehyde-3-phosphate dehydrogenase (GAPDH) (forward: 5’- GGGCTGCTTTTAACTCTGGC − 3’; reverse: 5’- TCTCGCTCCTGGAAGATGGT − 3’). All the genes were amplified in separate wells in triplicate. Gene expression was calculated using the 2^−△△CT^ method.

### Western blotting

Corneas were crushed in liquid nitrogen. RIPA Lysis Buffer (Beyotime, China) was used to extract the protein. The proteins were separated by 10% sodium dodecyl sulfate polyacrylamide Tris-glycine gels. The proteins in the gels were transferred to polyvinylidene difluoride membrane. The membrane was cropped according to the position of proteins’ color marker. Then the membranes were blocked with 5% milk in TBS for 2 h and then incubated overnight at 4 °C with the following primary antibodies: VEGF (1:1000, Abcam), ERK1/2 (1:1000, Invitrogen), Akt (1:3000: Abnova) and GAPDH (1:1000, Abcam). After 3 washes with TBST (TBS + 20% Tween-20), the membranes were incubated for 1 h at room temperature with goat anti-mouse (1:3000, Affinity Biosciences) and goat anti-rabbit (1:3000, Affinity Biosciences) secondary antibodies. ECL reagent was used to detect the immunological complexes membranes. And the proteins’ bands from the membranes were imprinted on the film in the dark room. The band intensities were measured by ImageJ software. The results were normalized to the expression of GAPDH.

### Statistical analysis

Statistical analysis was conducted using *SPSS 26.0* statistical software (SPSS Inc., Chicago, IL, USA). The mean and standard deviation are used to express all measurements (SD). All of the data in the preceding sections were analyzed using one-way ANOVA. We utilized either the least significant difference test (LSD-t) or Tamhane’s T2 for pairwise comparisons within the group. A *P* value < 0.05 was considered statistically significant.

## Results

### The expression of Scg3 could affect the growth of HUVECs in vitro

**Fig. 1:**
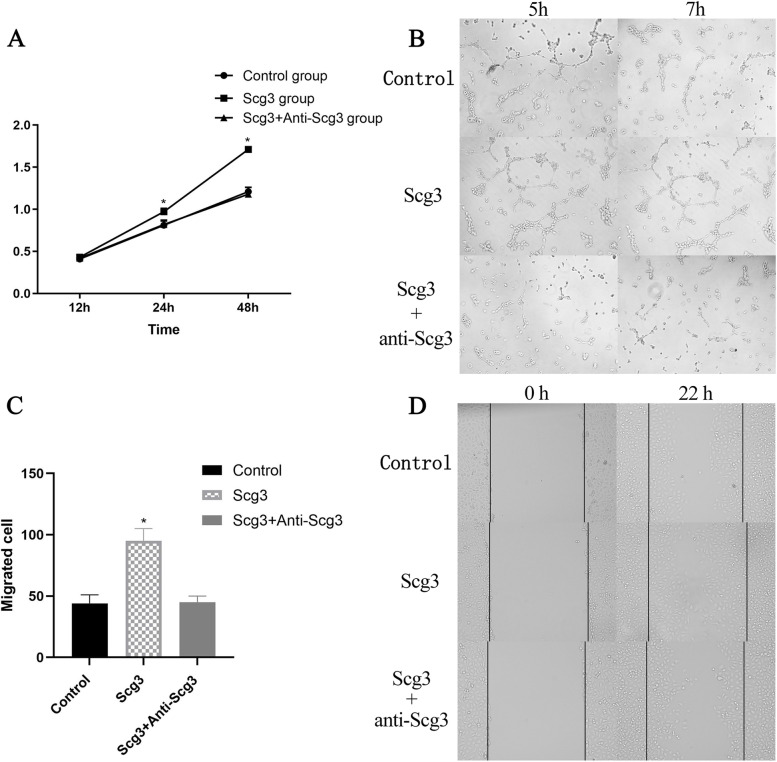
In vitro study. **A** A CCK8 assay was used to assess the proliferation of HUVECs stimulated with Scg3 antibodies for 12, 24 and 48 h. After 24 h, the proliferation of HUVECs stimulated by Scg3 antibodies was significantly lower than that of the other 2 groups. **B** In the tube formation assay, after 7 h, Scg3 antibodies (1 µg/ml Scg3 and 2 µg/ml anti-Scg3) significantly inhibited the angiogenesis of vascular endothelial cells compared with the Scg3 and control treatments alone. **C** In the wound healing migration assay, HUVECs were cultured in different media. The number of cells that migrated into the denuded area was quantified. After statistical analysis by one-way ANOVA, the cell proliferation in the Scg3 antibody mixture group was significantly lower than that in the Scg3 group, and there was no significant difference between the Scg3 group and the control group. **D** Representative images of HUVECs before and after migration. (*P < 0.05)

In the cell proliferation assay, Scg3 was found to promote endothelial cell proliferation, and the Scg3 antibody mixture inhibited Scg3-induced endothelial cell proliferation (Fig. 1 A). Furthermore, wound healing migration and tube formation assays were performed to assess HUVEC proliferation and angiogenesis, and the Scg3 antibody was found to inhibit endothelial cell proliferation and angiogenesis (Fig. 1B, C, D).

### Clinical observations

**Fig. 2:**
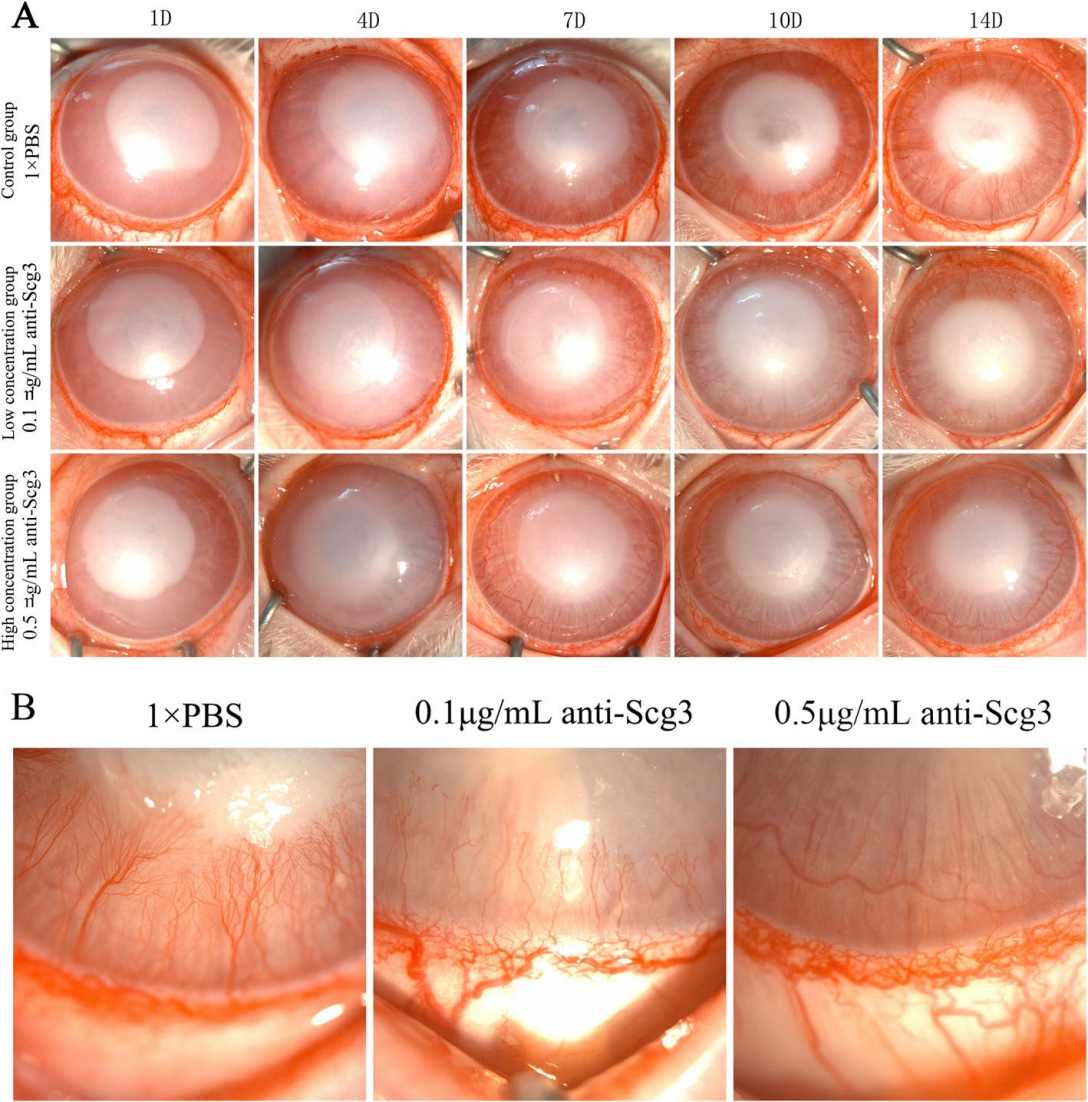
Slit lamp biomicrographs. Preoperative and postoperative observation of New Zealand rabbits. **A** One day after alkali burn injury, the boundary was clear in all 3 groups. Seven days after alkali burn injury, corneal neovascularization (CNV) started to appear in the corneal limbal in the two groups (control group and low concentration group). The diameter of the burn wound was reduced 10 days after alkali burn injury in the high concentration group. CNV grew to a large extent 14 days after alkali burn injury in the other two groups. **B** Fourteen days after alkali burn injury, there was obvious CNV growth into the corneal limbus in the control group. In the low concentration group, the CNV in the corneal limbus was slightly sparser than that in the control group. There was no obvious CNV growth during the observation period 14 days after alkali burn injury

**Fig. 3:**
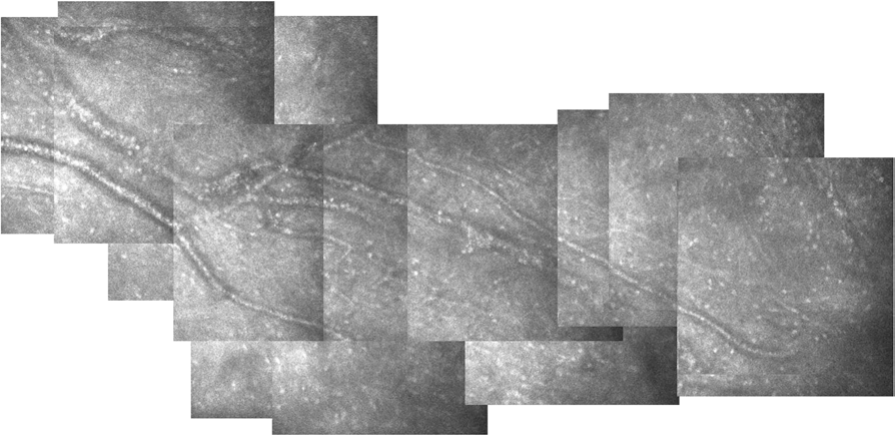
HRT III image, magnification 400 × 400 μm. Neovascularization was observed in the corneal stroma

After 14 days of subconjunctival injection with different agents, corneal neovascularization occurred in both the control group and the low concentration group (0.1 µg/ml Scg3 antibody). No corneal neovascularization was observed in the high concentration group (0.5 µg/ml Scg3 antibody). The diameter of the burn wound was significantly reduced when compared to the control group (Fig. 2 A & B). A large amount of active neovascularization was observed in the limbal of the control group by confocal corneal microscopy (Fig. 3).

### Immunofluorescence

**Fig. 4:**
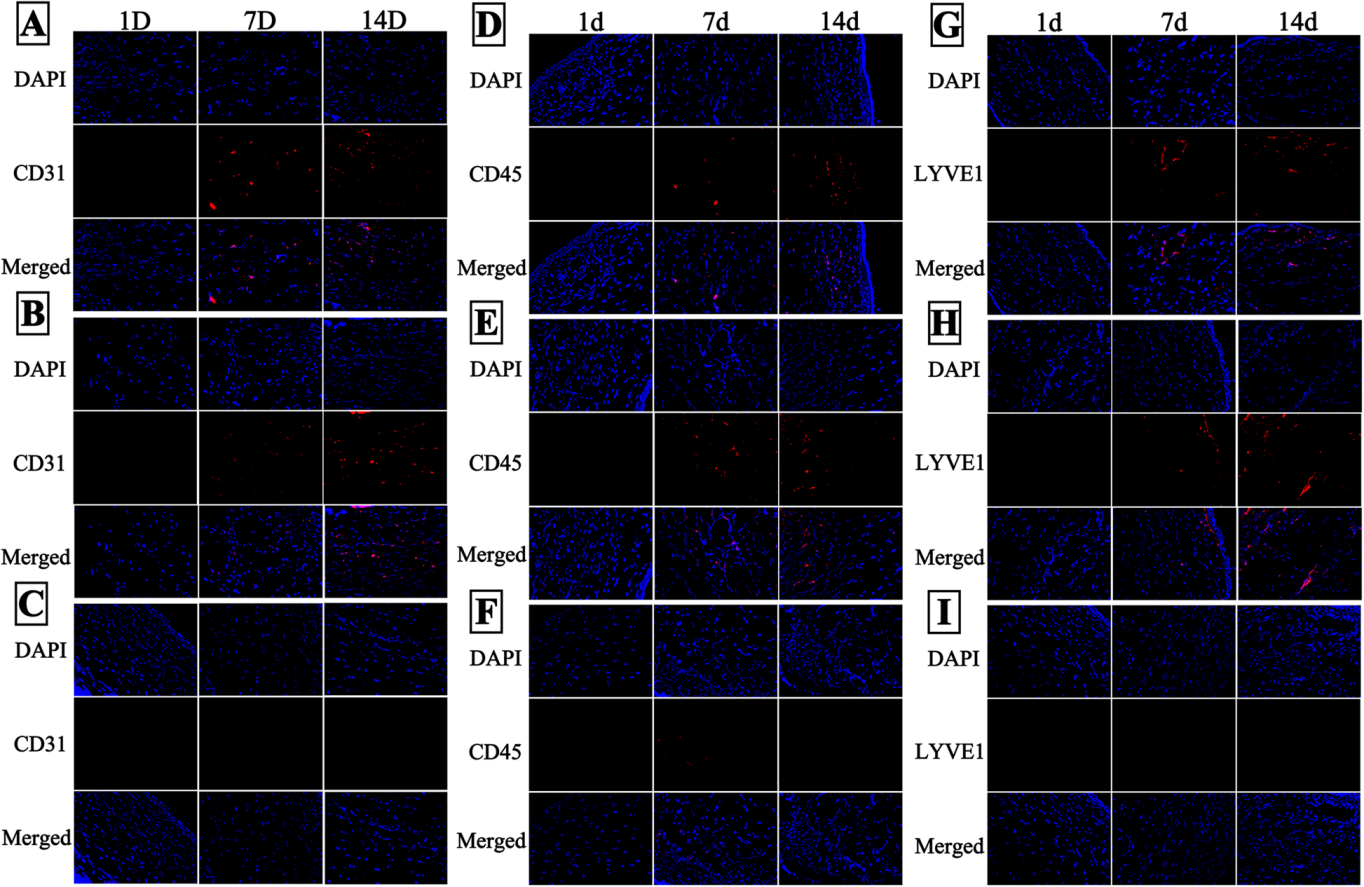
Representative images showing CD31, CD45 and LYVE1 expression in rabbit corneas. In the control group and the low-concentration group, the expression of all 3 targets (CD31, CD45 and LYVE1) was measured at 7 days. At 14 days, the expression of these 3 targets was significantly increased. In the high concentration group, weak expression of CD45 was only observed at 7 days. CD31 and LYVE1 were not expressed within 14 days. A, D & G represents the control group, B, E & H represents the low-concentration group, and C, F & I represents the high-concentration group

Immunofluorescence detection was performed on rabbit corneas at various time points after alkali burn, and it was found that the CD31, LYVE1 and CD45 expression was significantly inhibited after treatment with the high concentration of Scg3 antibody compared with the control treatment (Fig. 4).

### qPCR and western blotting

**Fig. 5:**
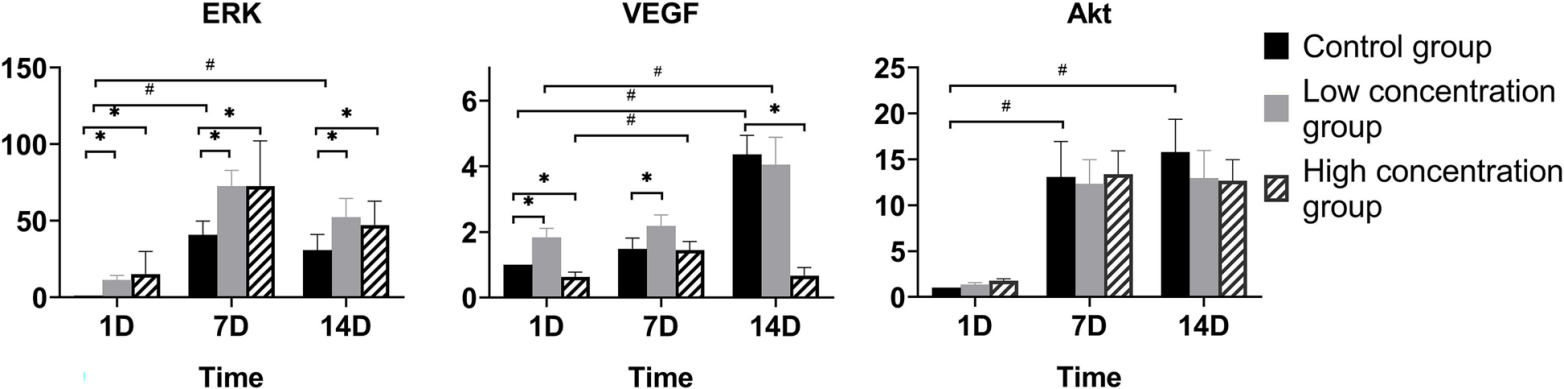
Time-dependent changes in the qPCR analysis. Significant differences were observed when comparing the three groups at the same time point (^*^P < 0.05) and the same group at the different time points (^#^*P < 0.05*). The levels of ERK in the low concentration group and high concentration group increased compared with those in the control group at all time points (*P*_*low−1D*_*=0.042, P*_*high−1D*_*=0.001, P*_*low−7D*_*=0.018, P*_*high−7D*_*=0.001, P*_*low−14D*_*=0.047, P*_*high−14D*_*=0.019*). The levels of ERK were significantly increased in all the groups after 7 days (*P*_*control−7D*_*=0.001, P*_*control−14D*_*=0.004, P*_*low−7D*_*=0.001, P*_*low−14D*_*=0.002, P*_*high−7D*_*=0.001, P*_*high−14D*_*=0.035*). The levels of VEGF in the low concentration group were significantly increased compared with those in the control group at 1 day and 7 days (*P*_*1D*_ *= 0.001, P*_*7D*_ *= 0.033*). The levels of VEGF in the control group were higher than those in the high concentration group at 1 day and 14 days (*P*_*1D*_ *= 0.046, P*_*7D*_ *= 0.001*). The levels of VEGF in the high concentration group decreased significantly at 14 days (*P = 0.005*). However, in the other two groups, the levels of VEGF increased at 14 days (*P*_*control*_*=0.001, P*_*low*_*=0.003*). In all three groups, the levels of Akt increased 7 days after alkali burn injury (*P*_*control−7D*_*=0.001, P*_*control−14D*_*=0.001, P*_*low−7D*_*=0.001, P*_*low−14D*_*=0.001, P*_*high−7D*_*=0.001, P*_*high−14D*_*=0.002*).

The qPCR analysis revealed that the levels of ERK raised at all time points in the low and high concentration groups. The levels of ERK were significantly increased in all the groups after 7 days, and the trends in each group were similar. The levels of VEGF in the control groups and the low concentration group were significantly increased at all the time points. The levels of VEGF in the high concentration group remained low at all the time points and decreased significantly at 14 days. In all three groups, the levels of Akt increased 7 days after alkali burn injury. There were no marked between-group differences in any of the groups (Fig. 5).

**Fig. 6:**
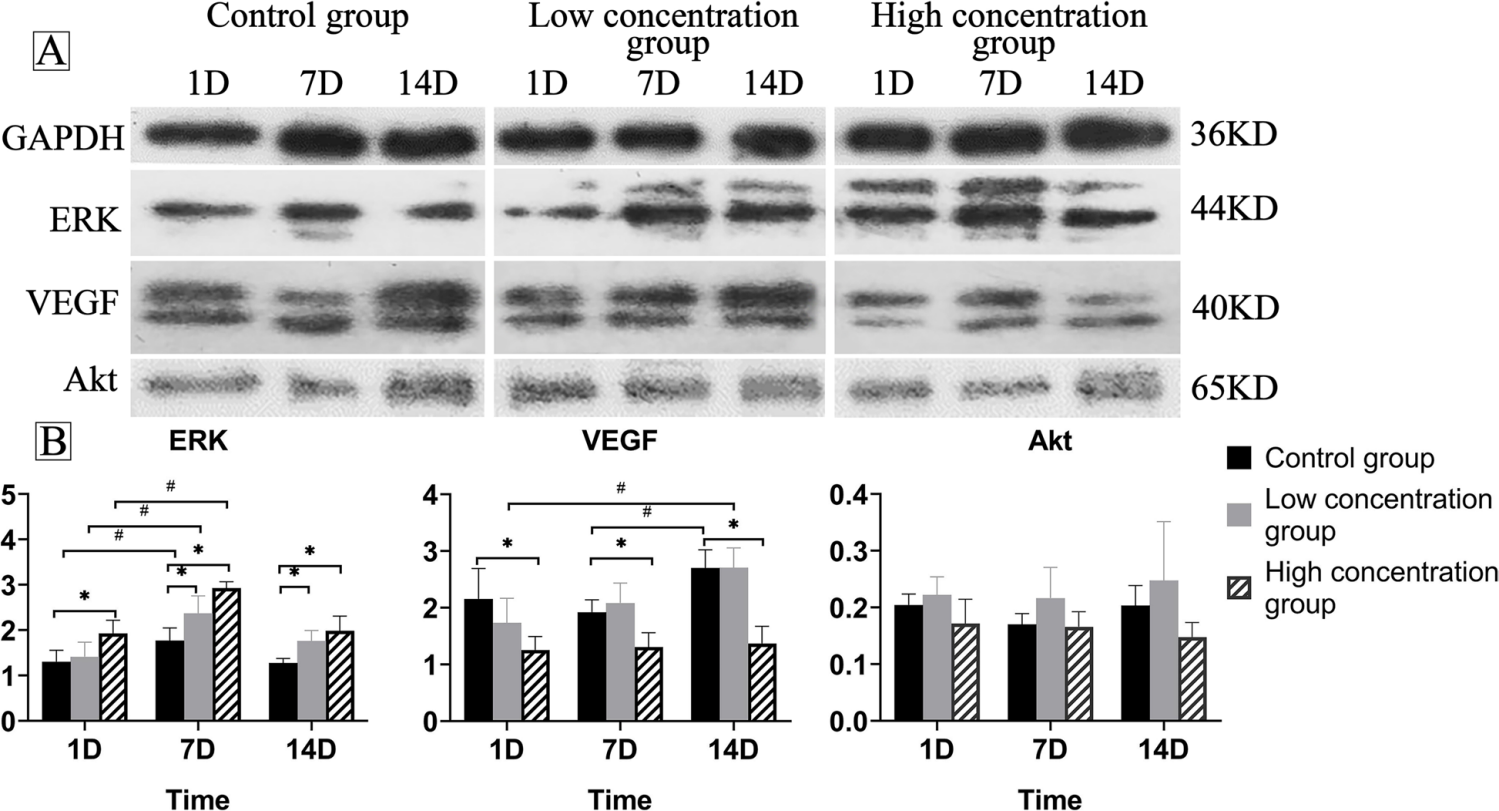
Time-dependent changes in the western blotting analysis. (**A**) Representative images showing ERK, VEGF and Akt expression in the different groups. **B** Significant differences were detected when comparing the three groups at the same time point (^*^P < 0.05) and the same group at the different time points (^#^*P < 0.05*). The levels of ERK in the high concentration group were higher than those in the control group at all the time points (*P*_*1D*_ *= 0.039, P*_*7D*_ *= 0.003, P*_*14D*_ *= 0.011*). The levels of ERK in the low concentration group were higher than those in the control group at 7 days and 14 days (*P*_*7D*_ *= 0.043, P*_*14D*_ *= 0.048*). Moreover, the levels of ERK were significantly increased in all the groups at 7 days (*P*_*control*_*=0.044, P*_*low*_*=0.020, P*_*high*_*=0.004*). The levels of VEGF in the control group and low concentration group were significantly increased at 14 days (*P*_*control*_*=0.047, P*_*low*_*=0.020*). In all three groups, the levels of Akt were not significantly different at any time point (all *P > 0.05*)

In the western blotting analysis, the low concentration and high concentration groups had significantly greater ERK levels than the control group at 7 and 14 days. The levels of ERK were significantly increased in all the groups at 7 days. At all-time points, the levels of VEGF in the control and low concentration groups were considerably higher than those in the high concentration group. The levels of VEGF in the high concentration group remained low for 14 days. In all three groups, the levels of Akt were not significantly different at any time point (Fig. 6 & Figure supplementary 1).

## Discussion

The present study assessed the effect of anti-Scg3 on human umbilical vein endothelial cells (HUVECs) and an animal model of corneal neovascularization (CNV) in vivo. In the in vitro study, anti-Scg3 inhibited the growth of HUVECs. In the in vivo study, subconjunctival injection of anti-Scg3 could interfere with the formation of CNV. We also found that the mitogen-activated protein kinase kinase (MEK)/extracellular signal-regulated kinase (ERK) pathway was involved in Scg3-induced proangiogenic intracellular signaling. These findings suggest that anti-Scg3 could influence the CNV process through the MEK/ERK pathway.

In the in vitro study, the cell proliferation assay, wound healing migration assay and tube formation assay showed that anti-Scg3 exerted an inhibiting effect on HUVECs. Anti-Scg3 not only affected HUVEC proliferation but also affected HUVEC angiogenesis and migration. We also observed Scg3 expression in the rabbit CNV model by western blotting. We further explored the optimal concentration of Scg3 antibody for the treatment of CNV by subconjunctival injection in a preliminary experiment. The preliminary results showed that if the Scg3 antibody concentration was too high (1 µg/mL or 1.5 µg/mL), it would cause a severe inflammatory response in the ocular surface and stimulate the growth of CNV; however, if the Scg3 antibody concentration was too low (0.02 µg/mL or 0.05 µg/mL), the growth of CNV might not be inhibited. Thus, we ultimately selected the concentrations of 0.1 µg/mL and 0.5 µg/mL Scg3 antibodies for this study. Moreover, considering the stimulation of subconjunctival injection, we treated CNV with eye drops containing different concentrations of Scg3 antibodies or reduced the number of subconjunctival injections, but neither of these approaches effectively inhibited the growth of CNV in the rabbit model. Therefore, we chose to administer Scg3 antibodies by subconjunctival injection to treat CNV, and then, the effect of Scg3 antibodies on corneal neovascularization was evaluated.

In the in vivo study, we observed the same inhibitory effect of anti-Scg3 on the CNV animal model as we observed in the in vitro study. Scg3 antibody efficiently inhibited CNV at 7 days after corneal alkali burn injury. In our previous study, with Scg3 antibody interference, there was a statistically significant difference in CNV length and CNV areas. The expression levels of inflammatory factors (ICAM-1, IL-10 and TGF-β1) from animal tears’ were also decreased at 7 days postoperatively [23]. The Scg3 antibody could inhibit the CNV progress and reduce the ocular surface inflammatory response after corneal alkali burn in rabbits. Because these inflammatory cytokines results were from ocular surface. In this study, we further measured the expression of CD31, CD45 and LYVE1 in the cornea. The CD31 plays an important role in the regulation of angiogenesis and vascular permeability [24]. And the CD31 served as a specific endothelial cell marker to detect neovascularization [25]. The CD45 is a leukocyte’s marker to indicate leukocyte infiltration [26]. The corneal inflammation could assess by CD45 explanation. The corneal lymphangiogenesis following injury were evaluated by the lymphatic vascularized marker LYVE1 [27]. And the CNV always occurs simultaneously with lymph angiogenesis [28]. We found the vascular CD31, lymphatic LYVE1 and leukocyte’s CD45 expression was significantly inhibited by the high concentration (0.5 µg/mL) of Scg3 antibody. Since the Scg3 antibody inhibiting inflammatory response in cornea, the CNV angiopoiesis and growth was restrained. These findings imply that Scg3 may play a role in the etiology of CNV. Scg3 was identified as an angiogenic ligand related with illness in Li’s study [18]. The normal cornea is a transparent tissue with no vessels or lymph. Anti-Scg3 might not influence normal corneal tissue but only affect CNV. Therefore, anti-Scg3 injection could be developed for clinical use in future studies.

In the subsequent qPCR and western blotting assays, we found that Scg3 might function via a different signalling pathway from VEGF. Scg3 activates ERK but not Akt, whereas VEGF causes phosphorylation of ERK and Akt [18]. These findings imply that the intracellular signaling pathways of Scg3 and VEGF, which originate from separate receptors, may partially converge to regulate shared angiogenic activities. MEK/ERK is one of the important pathways in the formation of corneal neovascularization (CNV) and is widely present in eukaryotic cells [29]. When the cornea is stimulated by the inflammatory response, MEK in this pathway can be activated by a large number of inflammatory factors [30]. The downstream ERK protein receives the activation signal, induces phosphorylation and then starts to regulate vascular endothelial cells through downstream signalling molecules in the cytoplasm and nucleus [31]. Downstream signaling cascades and cellular responses are determined by ERK1/2 subcellular localization [32]. After vascular endothelial cells receive molecular signals, matrix metalloproteinases (MMPs), such as MMP-2, MMP-9, and MMP-14, are activated, and then, these proteins start to rebuild the vascular basement membrane, which is conducive to the outward expansion and proliferation of vascular endothelial cells [31, 33]. Additionally, phosphorylation drives the further differentiation of vascular endothelial cells to form lumen structures and eventually form new vessels [31, 33]. Vascular endothelial growth factor receptor (VEGFR) 1, 2 and 3 can activate multiple intracellular signaling pathways, including the MEK/ERK and Akt and Stat3 pathways [34]. In VEGF-induced angiogenesis, the MEK/ERK pathway plays a crucial role [35, 36]. Furthermore, MER/ERK suppression inhibits VEGF-induced endothelial proliferation [37]. The result of this preliminary study suggest that the Scg3 activate the MEK/ERK pathway. Scg3, like VEGF, can activate a variety of intracellular signaling pathways. This preliminary study will help provide an in-depth understanding of the molecular mechanisms underlying the effects of Scg3 and facilitate the development of new anti-Scg3 therapeutic strategies by blocking its downstream intracellular signaling cascades.

The limitations of the current research should be taken into account. First, subconjunctival injection activated CNV to some extent. Second, more targets related to CNV need to be identified to evaluate the influence of anti-Scg3. Finally, various receptors can activate the MEK/ERK pathway [38]. And which signaling pathway includes the Scg3 receptor and the molecular mechanisms by which Scg3 functions require further study.

## Conclusion

We found that Scg3 antibodies can effectively inhibit the formation of CNV, which may be related to the fact that Scg3 antibodies can inhibit the inflammatory response after corneal alkali burn injury. And we will further study the mechanism by which Scg3 is involved in corneal neovascularization and lymphatic formation in vivo and provide new strategies for the treatment or combined treatment of CNV.

## Supplementary Information

**Additional file 1. **Representative raw images showing ERK, VEGF andAkt expression in the different groups.

## Data Availability

The datasets used and/or analysed during the current study available from the corresponding author on reasonable request.

## Abbreviations

CNV: Corneal neovascularization
HUVEC: Human umbilical vein endothelial cells
LYVE1: lymphatic vessel endothelial hyaluronan receptor 1
qPCR: Quantitative Real-time PCR
VEGF: Vascular endothelial growth factor
VEGFR: Vascular endothelial growth factor receptor
ERK: Extracellular signal-regulated kinase
GAPDH: Glyceraldehyde-3-phosphate dehydrogenase
MEK/ERK: Mitogen-activated protein kinase kinase /Extracellular signal-regulated kinase
