# Supplementary material for: Inhibitory effect of anti-Scg3 on corneal neovascularization: a preliminary study
Source: BMC Ophthalmol. 2022 Nov 28;22:455. doi: 10.1186/s12886-022-02690-7 (PMC9703748; doi:10.1186/s12886-022-02690-7)
Supplement: Supplementary file 1 — Additional file 1. Representative raw images showing ERK, VEGF andAkt expression in the different groups. [file 12886_2022_2690_MOESM1_ESM.pdf]

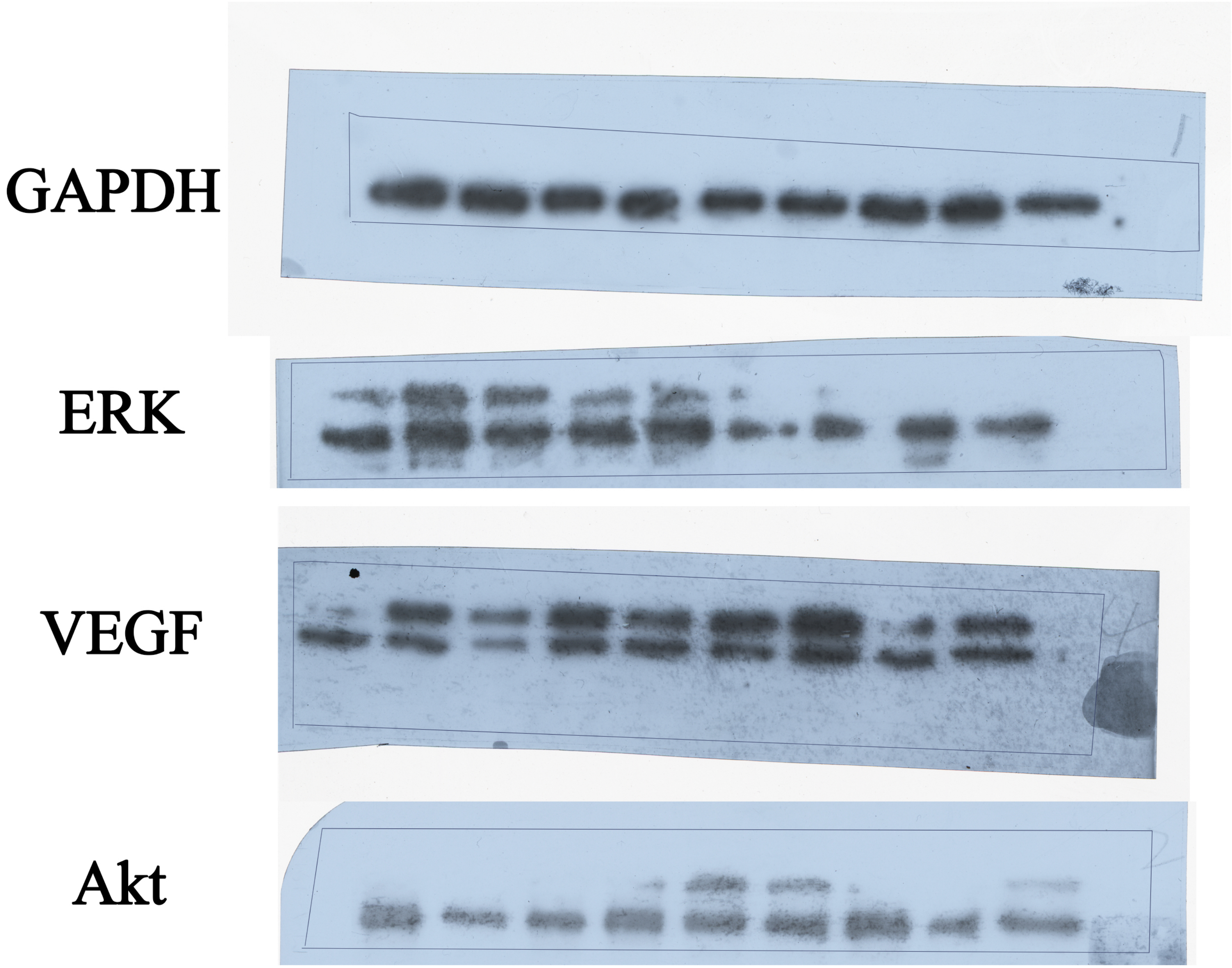

Representative raw images showing ERK, VEGF and Akt expression in the different groups.
